# Supplementary material for: The SARS-CoV-2-Inactivating Activity of Hydroxytyrosol-Rich Aqueous Olive Pulp Extract (HIDROX®) and Its Use as a Virucidal Cream for Topical Application
Source: Viruses. 2021 Feb 2;13(2):232. doi: 10.3390/v13020232 (PMC7913061; doi:10.3390/v13020232)
Supplement: Supplementary file 1 [file viruses-13-00232-s001.pdf]

Supplementary Materials

Table S1. Information of recombinant proteins and antibodies

Recombinant proteins	First antibody for WB	Secondary antibody for WB
SARS-CoV-2 (2019-nCoV) Spike S1-His Recombinant Protein [Catalog No. 40591-V08H][*]	SARS-CoV-2/2019-nCoV Spike Antibody, Rabbit PAb [Catalog No. 40591][*]	Mouse anti-rabbit IgG peroxidase conjugate [Catalog No. A1949][‡]
Recombinant 2019-nCoV Spike Protein (RBD, mFc Tag) [Catalog No. PKSR030500][†]	SARS-CoV-2 Spike RBD Polyclonal Antibody [Catalog No. E-AB-V1006][†]	
SARS-CoV-2 (2019-nCoV) Spike Protein (S2 ECD, His tag) [Catalog No. 40590-V08B][*]	SARS-CoV-2/2019-nCoV Spike/S2 Antibody, Rabbit PAb [Catalog No. 40590-T62][*]	
SARS-CoV-2 (2019-nCoV) Nucleocapsid Protein (His tag) [Catalog No. 40588-V08B][*]	SARS-CoV-2 (2019-nCoV) Nucleoprotein/NP Antibody, Rabbit Mab [Catalog No. 40143-R019][*]	

[*]Purchased from Sino Biological Inc., Beijing, China

[†]Purchased from Elabscience Biotechnology Inc., TX, USA

[‡]Purchased from Sigma-Aldrich, Inc., Saint Louis, MO, USA

The data presented in this study

Figure 1A

	0.5 h					1 h			
			HIDROX					HIDROX	
	PBS	4.5 mg/ml	0.9 mg/ml	0.45 mg/ml		PBS	4.5 mg/ml	0.9 mg/ml	0.45 mg/m
tube 1	6.25	4.25	6.25	5.75		7.25	2.75	5.25	5.75
tube 2	6.75	3.75	5.75	6.25		6.75	2.25	4.75	5.75
tube 3	7.25	4.75	5.25	6.25		6.75	3.25	5.25	6.25
mean	6.75	4.25	5.75	6.083333		6.916667	2.75	5.083333	5.916667
stdev	0.5	0.5	0.5	0.288675		0.288675	0.5	0.288675	0.288675
PBS group − test group		2.5	1	0.666667			4.166667	1.833333	1
Virus inactivation rate		99.68377	90	78.45565			99.99319	98.5322	90

	3 h					6 h			
		HIDROX					HIDROX		
	PBS	4.5 mg/ml	0.9 mg/ml	0.45 mg/ml		PBS	4.5 mg/ml	0.9 mg/ml	0.45 mg/m
tube 1	6.25	2.25	3.75	5.25		6.25	2.25	3.25	4.25
tube 2	6.25	2.25	3.25	5.25		5.75	2.25	3.25	4.75
tube 3	6.75	2.25	3.75	5.75		6.25	2.25	3.25	5.25
mean	6.416667	2.25	3.583333	5.416667		6.083333	2.25	3.25	4.75
stdev	0.288675	0	0.288675	0.288675		0.288675	0	0	0.5
PBS group − test group		4.166667	2.833333	1			3.833333	2.833333	1.333333
Virus inactivation rate		99.99319	99.85322	90			99.98532	99.85322	95.35841

	24 h			
		HIDROX		
	PBS	4.5 mg/ml	0.9 mg/ml	0.45 mg/ml
tube 1	6.25	2.25	2.25	3.75
tube 2	5.75	2.25	2.25	4.25
tube 3	6.25	2.25	2.25	3.75
mean	6.083333	2.25	2.25	3.916667
stdev	0.288675	0	0	0.288675
PBS group − test group		3.833333	3.833333	2.166667
Virus inactivation rate		99.98532	99.98532	99.31871

Figure 1B

			**5 min**			
				HIDROX		
			PBS	5.63 mg/m	11.25 mg/ml	
		tube 1	5.25	4.75	4.25	
		tube 2	5.75	4.25	4.25	
		tube 3	6.25	5.25	4.25	
		tube 4	5.25	4.75	4.25	
		mean	5.625	4.75	4.25	
		stdev	0.478714	0.408248	0	
PBS group - test group				0.875	1.375	
Virus inactivation rate				86.66479	95.78303	

Figure 2

	3 h			
	PBS	0.9 mg/ml HIDROX	0.9 mg/ml HT	0.05 mg/ml HT
tube 1	5.75	3.25	5.75	5.25
tube 2	5.75	3.25	4.25	5.25
tube 3	5.25	3.25	4.75	5.25
tube 4	5.75	3.75	5.25	5.25
mean	5.625	3.375	5	5.25
stdev	0.25	0.25	0.645497224	0
PBS group − test group		2.25	0.625	0.375
Virus inactivation rate		99.43765867	76.28626294	57.83034966
	24 h			
	PBS	0.9 mg/ml HIDROX	0.9 mg/ml HT	0.05 mg/ml HT
tube 1	5.75	≦2.25	≦2.25	4.75
tube 2	5.75	≦2.25	≦2.25	5.25
tube 3	6.25	≦2.25	≦2.25	5.25
tube 4	5.75	≦2.25	≦2.25	5.75
mean	5.875	≦2.25	≦2.25	5.25
stdev	0.25	0	0	0.40824829
PBS group − test group		≧3.625	≧3.625	0.625
Virus inactivation rate		≧99.976	≧99.976	76.28626294

Figure 3A

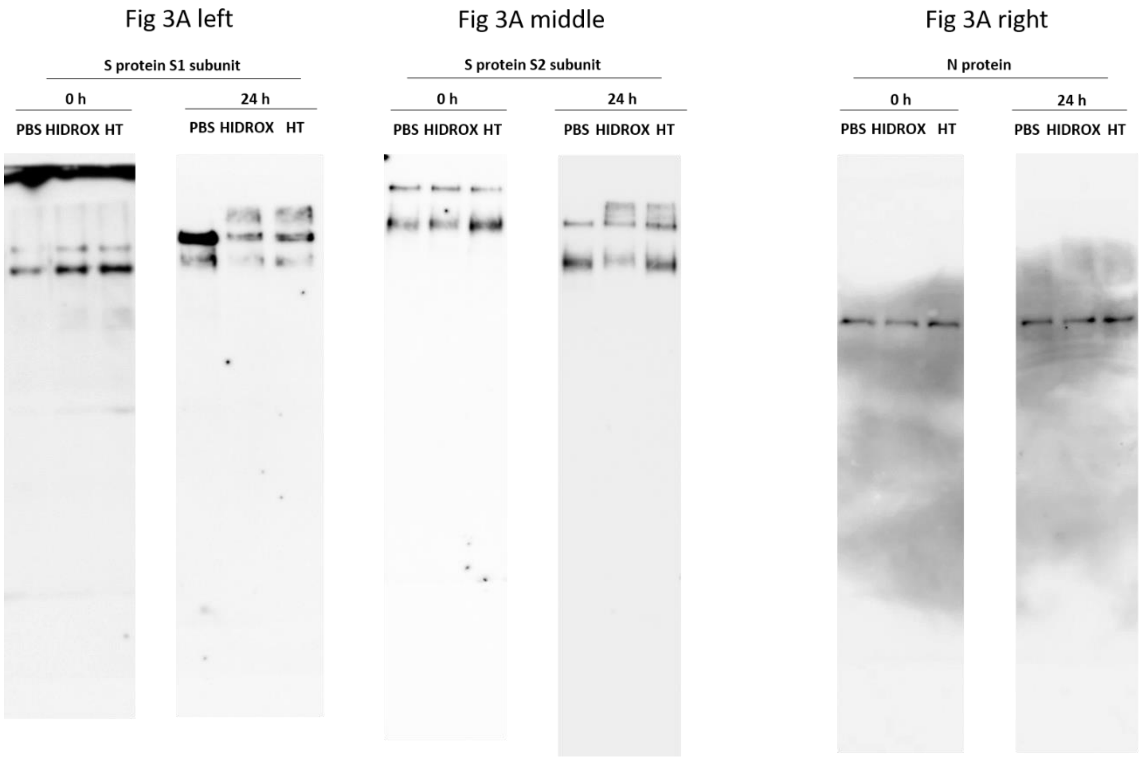
Fig 3A left
S protein S1 subunit
0 h
24 h
PBS HIDROX HT
PBS HIDROX HT
Fig 3A middle
S protein S2 subunit
0 h
24 h
PBS HIDROX HT
PBS HIDROX HT
Fig 3A right
N protein
0 h
24 h
PBS HIDROX HT
PBS HIDROX HT

Figure 3B

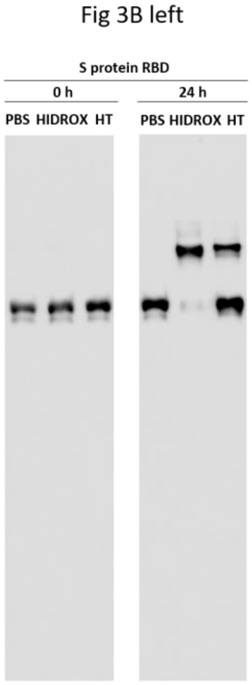

Fig 3B left
S protein RBD
0 h
24 h
PBS HIDROX HT
PBS HIDROX HT

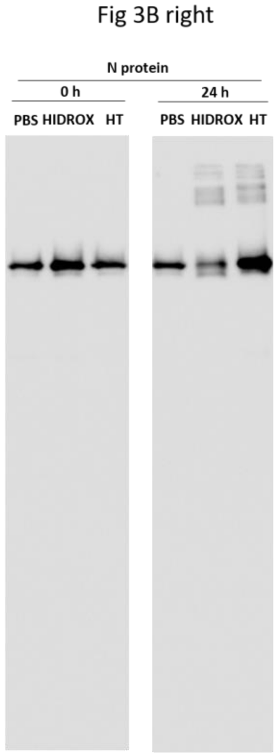

Fig 3B right
N protein
0 h
24 h
PBS HIDROX HT
PBS HIDROX HT

Figure 4

Fig 4 left

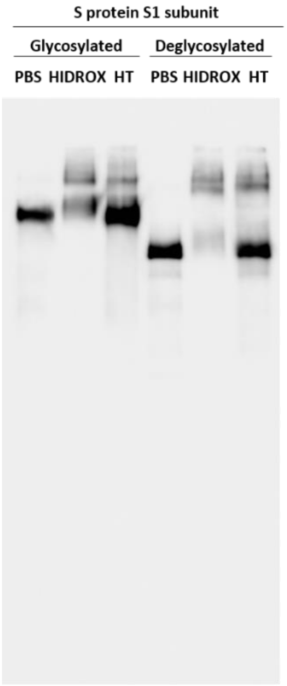

Fig 4 middle

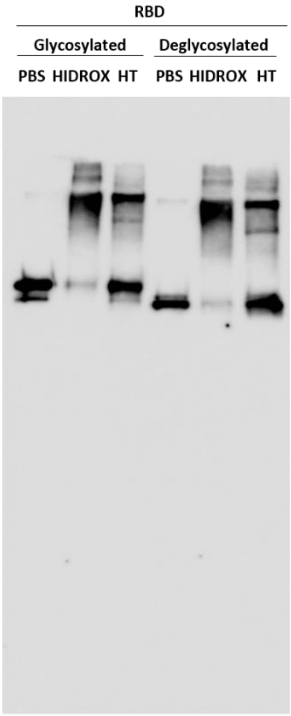

Fig 4 right

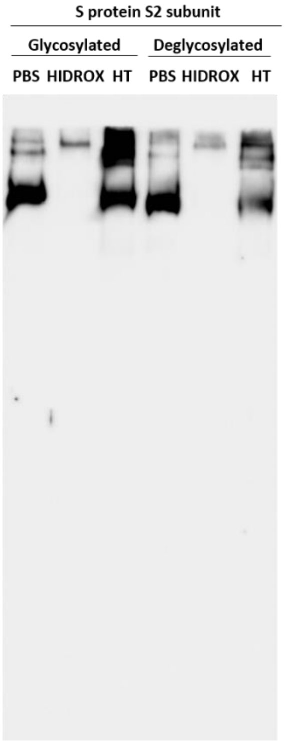

Figure 5

			**0 h**		
			PBS	HIDROX (0.9 mg/ml)	HT (0.9 mg/ml)
		Tube 1	24.798	24.566	24.092
		Tube 2	26.531	23.468	23.949
		Tube 3	23.487	24.589	25.082
		Tube 4	24.495	24.384	23.903
		mean	24.82775	24.25175	24.2565
		STDEV	1.266265	0.530485548	0.55618612
PBS group − test group				0.576	0.57125
			**24 h**		
			PBS	HIDROX (0.9 mg/ml)	HT (0.9 mg/ml)
		Tube 1	23.868	26.611	26.921
		Tube 2	23.505	27.218	27.24
		Tube 3	23.457	26.844	26.154
		Tube 4	25.002	27.922	26.021
		mean	23.958	27.14875	26.584
		STDEV	0.719779	0.572933606	0.590416237
PBS group − test group				−3.19075	−2.626

Figure 6

10 min	HIDROX			
	0%	2%	5%	10%
tube 1	5.25	4.75	4.75	4.25
tube 2	4.25	4.75	4.25	3.75
tube 3	5.25	4.75	4.75	3.75
tube 4	4.75	4.75	4.25	3.25
tube 5	6.25	4.25	4.25	3.75
tube 6	5.25	4.25	4.25	3.75
tube 7	4.75	5.25	4.75	3.75
tube 8	4.75	4.25	3.75	4.25
mean	5.0625	4.625	4.375	3.8125
stdev	0.593867	0.353553	0.353553	0.320435
0% HIDROX group − test group		0.4375	0.6875	1.25
Virus inactivation rate		63.48259	79.46475	94.37659

30 min	HIDROX			
	0%	2%	5%	10%
tube 1	5.25	3.75	2.75	≦1.25
tube 2	4.75	3.75	2.25	2.75
tube 3	5.75	3.75	2.75	2.75
tube 4	5.25	4.25	3.75	≦1.75
tube 5	4.25	4.75	4.25	3.25
tube 6	5.25	3.75	3.75	3.25
tube 7	6.25	3.75	3.75	3.25
tube 8	5.25	4.25	3.75	3.75
	5.25	4	3.375	≦2.75
	0.597614	0.377964	0.694365	0.3763863
		1.25	1.875	≧2.5
		94.37659	98.66648	≧99.684

1 h	HIDROX			
	0%	2%	5%	10%
tube 1	5.25	3.25	3.25	≦1.25
tube 2	5.25	2.75	≦1.75	≦1.25
tube 3	4.75	3.25	2.25	≦1.25
tube 4	6.25	2.75	≦1.25	≦1.75
tube 5	5.25	4.25	2.75	2.25
tube 6	5.25	4.25	2.25	≦1.75
tube 7	4.75	3.75	2.75	2.75
tube 8	5.25	3.75	2.25	2.25
	5.25	3.5	≦2.3125	≦1.8125
	0.46291	0.597614	0.408248	0.288675
		1.75	≧2.9375	≧3.4375
		98.22172	≧99.885	≧99.964

3 h	HIDROX			
	0%	2%	5%	10%
tube 1	5.75	≦1.25	≦1.25	≦1.25
tube 2	5.25	≦1.25	≦1.25	≦1.25
tube 3	5.25	≦1.25	≦1.25	≦1.25
tube 4	5.75	≦1.25	≦1.25	≦1.25
tube 5	4.75	2.25	≦1.25	≦1.25
tube 6	4.75	2.25	≦1.25	≦1.25
tube 7				
tube 8				
mean	5.25	≦1.5833	≦1.25	≦1.25
stdev	0.447214	0.516398	0	0
0% HIDROX group − test group		≧3.667	≧4	≧4
Virus inactivation rate		≧99.979	≧99.99	≧99.99

6 h	HIDROX			
	0%	2%	5%	10%
tube 1	4.75	≦1.25	≦1.25	≦1.25
tube 2	5.25	≦1.25	≦1.25	≦1.25
tube 3	5.25	≦1.25	≦1.25	≦1.25
tube 4	4.25	≦1.25	≦1.25	≦1.25
tube 5				
tube 6				
tube 7				
tube 8				
	4.875	≦1.25	≦1.25	≦1.25
	0.478714	0	0	0
		≧3.625	≧3.625	≧3.625
		≧99.976	≧99.976	≧99.976
